# Potent Effects of Flavonoid-Rich Extract from *Rosa laevigata* Michx Fruit against Hydrogen Peroxide-Induced Damage in PC12 Cells via Attenuation of Oxidative Stress, Inflammation and Apoptosis

**DOI:** 10.3390/molecules190811816

**Published:** 2014-08-07

**Authors:** Min Liu, Youwei Xu, Xu Han, Chen Liang, Lianhong Yin, Lina Xu, Yan Qi, Yanyan Zhao, Jinyong Peng, Changkai Sun

**Affiliations:** 1 College of Pharmacy, Dalian Medical University, Western 9 Lvshunnan Road, Lvshunkou District, Dalian 116044, China; 2 College of Basic Medical Sciences, Dalian Medical University, Western 9 Lvshunnan Road, Lvshunkou District, Dalian 116044, China; 3 Research Institute of Integrated Traditional and Western Medicine of Dalian Medical University, Dalian 116044, China; 4 Liaoning Provincial Key Laboratory of Brain Diseases and Institute of Medical Education, Western 9 Lvshunnan Road, Lvshunkou District, Dalian 110644, China

**Keywords:** apoptosis, flavonoid-rich extract, inflammation, oxidative stress, *Rosa laevigata* Michx

## Abstract

Oxidative stress-induced neuronal death has an important role in the pathogenesis of neurodegenerative disorders. The effects and mechanisms of action of the total flavonoids (TFs) from *Rosa laevigata* Michx fruit against hydrogen peroxide (H_2_O_2_)-induced oxidative injury in PC12 cells were investigated in this study. The results demonstrated that the TFs protected against cell apoptosis, DNA and mitochondrial damage caused by H_2_O_2_ based on single cell gel electrophoresis, *in situ* terminal deoxynucleotidyltransferase dUTP nick end labeling (TUNEL), flow cytometry and transmission electron microscope (TEM) assays. In addition, the TFs notably decreased cytochrome C release from mitochondria into the cytosol and intracellular Ca^2+^ levels, and diminished intracellular generation of reactive oxygen species (ROS). Furthermore, the TFs inhibited the phosphorylation levels of JNK, ERK and p38 MAPK as well as down-regulated the expressions of IL-1, IL-6, TNF-α, Fas, FasL, CYP2E1, Bak, caspase-3, caspase-9, p53, COX-2, NF-κB, AP-1, and up-regulated the expressions of Bcl-2 and Bcl-xl. In conclusion, these results suggest that the TFs from *R. laevigata* Michx fruit show good effects against H_2_O_2_-induced oxidative injury in PC12 cells by adjusting oxidative stress, and suppression of apoptosis and inflammation, and could be developed as a potential candidate to prevent oxidative stress in the future.

## 1. Introduction

Oxidative injury has been involved in the pathogenesis of some neurological diseases including Parkinson's disease, Alzheimer’s disease [1] and stroke [2]. It is well known that oxidation is an essential process in living organisms, however, excessive oxidation can cause lipid peroxidation, DNA and proteins oxidation, and promote cellular damage and death [3]. In addition, oxidative stress can enhance intracellular Ca^2+^ concentration [4], and activate neuro-inflammatory reactions [5] and apoptotic pathways. When these processes happen, different types of cells can generate lots of hydrogen peroxide (H_2_O_2_). Due to its high cellular membrane permeability, H_2_O_2_ is toxic to both the producing cells and neighboring ones [6], thus it is often used as a toxicant to mimic *in vitro* models of oxidative stress-induced injury. Furthermore, many studies have shown that H_2_O_2_-induced oxidative stress occurs through triggering mitochondrial dysfunction, which correlates with the changes of proteins in the Bcl-2 family, cytochrome C release and activation of caspases [7]. Mitogen-activated protein kinases (MAPKs) can regulate complicated signaling pathways related to cell growth and apoptosis [8]. These kinases can also change cellular signaling in the nucleus by activation of oxidation sensitive transcription factors, including activator protein-1 (AP-1) and nuclear factor-κappaB (NF-κB) [9]. Thus, therapeutic strategies aimed at blocking of reactive oxygen species (ROS)-induced apoptosis might be effective for the treatment of these diseases.

Traditional Chinese Medicines (TCMs) have been attracting more and more attention because of their high efficiency and low toxicity [10,11,12]. Nowadays, herbal extracts and natural products with antioxidant properties can significantly alleviate the diseases caused by oxidative stress [13]. Some natural products including quercetin [14], curcumin [15], caffeic acid [16] and resveratrol [17] have been shown to be neuroprotective against oxidative injury. Thus, it is reasonable to exploit effective natural products from medicinal plants for the treatment of oxidative damage.

*Rosa laevigata* Michx is a famous medicinal plant, and the fruit of this plant has been widely used in China for a long time [18]. Our previous studies have shown that the total flavonols (TFs) fraction from the fruit has potent antioxidant, hypolipidemic, hepatoprotective and antithrombotic activities through attenuating inflammation, suppression of apoptosis and altering MAPK signaling pathways [19]. In addition, the extract also shows protective effects against oxidative damage to human umbilical vein endothelial cells [20]. However, there are no papers to report the activities of the TFs against H_2_O_2_-induced PC12 cell injury.

The aim of the present work was to study the protective effects of the TFs from *R. laevigata* Michx fruit against H_2_O_2_-induced PC12 cell injury, and then the possible mechanisms were also explored. As we all know, H_2_O_2_ can destroy neurons by inducing apoptosis, which may perturb the cell’s natural antioxidant defence system, resulting in damage to implicated biological molecules and pathological processes. Thus, we hypothesized that the ROS-induced oxidative stress caused by H_2_O_2_ may lead to cell apoptosis, whereas the TFs could scavenge ROS and consequently reduce the apoptosis.

## 2. Results and Discussion

### 2.1. Effects of the TFs on H_2_O_2_-Induced Injury in PC12 Cells

**Figure 1:**
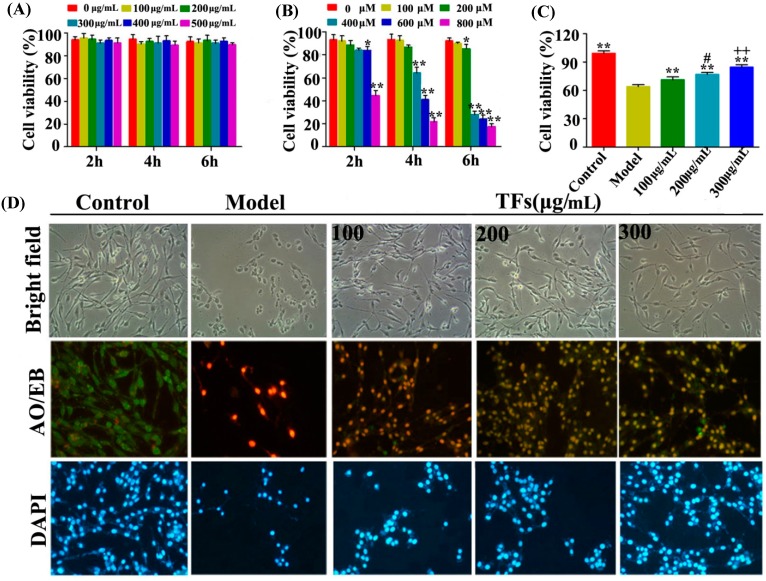
Protective effects of the TFs on H_2_O_2_-induced cell injury in PC12 cells. (**A**) Dose- and time-dependent effects of TFs on cell viability; (**B**) Dose- and time-dependent effects of H_2_O_2_ on cell viability; (**C**) Pretreatment with TFs (100, 200 and 300 μg/mL,1 h) alleviated H_2_O_2_-induced cell injury; (**D**) Morphological and Fluorescence images of PC12 cells stained by AO/EB and DAPI (100×, final magnification). Datas are presented as mean ± SD (*n* = 5). * *p* < 0.05 and ** *p* < 0.01 compared with model group. ^#^ *p* < 0.05 representes the comparison between 100 μg/mL and 200 μg/mL treatment groups, and ^++^ *p* < 0.01 representes the comparisons between 200 μg/mL and 300 μg/mL treatment groups.

The effects of TFs and H_2_O_2_ on cell viability were investigated first, and we found that the TFs in the 100–500 μg/mL range didn’t affect the viability of PC12 cells (Figure 1A), while H_2_O_2_ at the range of 400–800 μM significantly decreased cell viability in a dose- and time-dependent manner (Figure 1B). In the present paper, the average viability of the cells treated with H_2_O_2_ (400 μM, 4 h) was decreased to 66.1%, and TFs pretreatment (200 and 300 μg/mL) for 1 h showed significant effects against H_2_O_2_-induced cell injury (Figure 1C). In addition, obvious morphological changes were found in different groups using bright field images, AO/EB (acridine orange/ethidium bromide, the reagents used for staining assay to detect necrotic and apoptosis) and DAPI staining of PC12 cells. As shown in Figure 1D, the cell death and the numbers of apoptotic or necrotic cells gradually decreased when the concentrations of TFs increased. In addition, the nucleus was condensed and nuclear apoptotic bodies were formed as well when the cells were treated with H_2_O_2_, however, TFs significantly attenuated these changes.

### 2.2. Effects of the TFs on the Levels of H_2_O_2_-Induced ROS and Ca^2+^ Release

**Figure 2:**
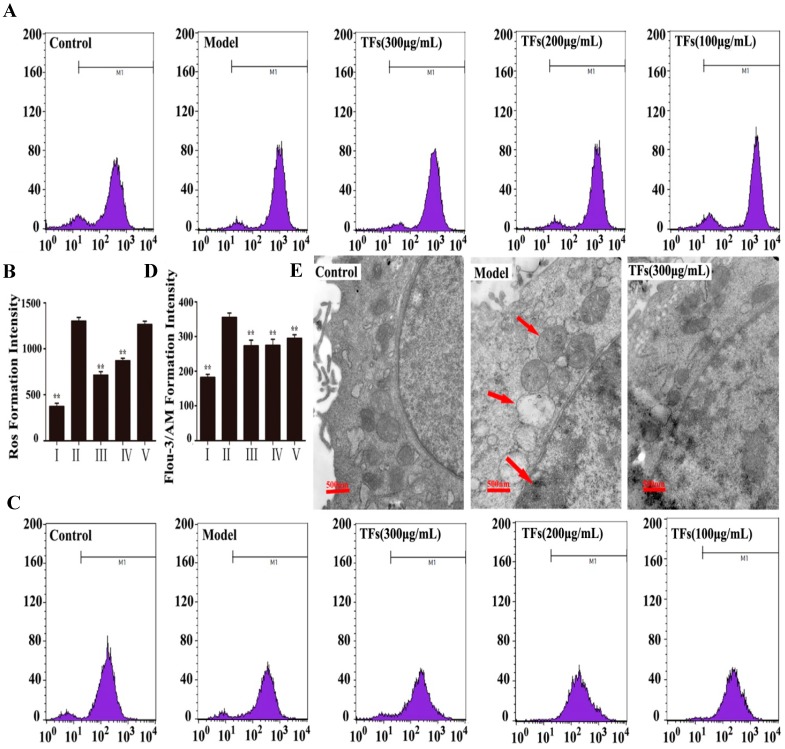
ROS generation detected by flow cytometry (**A**, **B**); The level of Ca^2+^ detected by flow cytometry (**C**, **D**); Protective effect of the TFs on the ultra-structure of PC12 cells (40,000×, final magnification) (**E**). Data are presented as mean ± SD (*n* = 5). * *p* < 0.05 and ** *p* < 0.01 compared with model group. The arrows pointed cytoplasmic vacuoles, chromatin condensation and mitochondrial swelling of the cells treated by H_2_O_2_.

The levels of ROS and Ca^2+^ were measured treatment with H_2_O_2_ for 4 h. As shown in Figure 2A,B, H_2_O_2 _markedly increased the ROS level in the model group, which was markedly decreased by the TFs (300 μg/mL). In addition, the level of Ca^2+^ in the model group was significantly increased by H_2_O_2_, and was decreased by the TFs, and there were no difference between the different doses (100–300 μg/mL) of the treatment groups (Figure 2C,D).

### 2.3. Effects of the TFs on Ultrastructure Changes Caused by H_2_O_2_

Transmission electron microscopy (TEM) was used to observe the ultrastructure of PC12 cells. As shown in Figure 2E, normal cells exhibited complete cell membranes and normal nuclei, but the cells treated by H_2_O_2_ appeared distinct, with cytoplasmic vacuoles, chromatin condensation and mitochondrial swelling as indicated by the arrows. Compared with the model group, the TFs meaningfully reversed the above conditions.

### 2.4. TFs Alleviated H_2_O_2_-Induced DNA Damage

**Figure 3:**
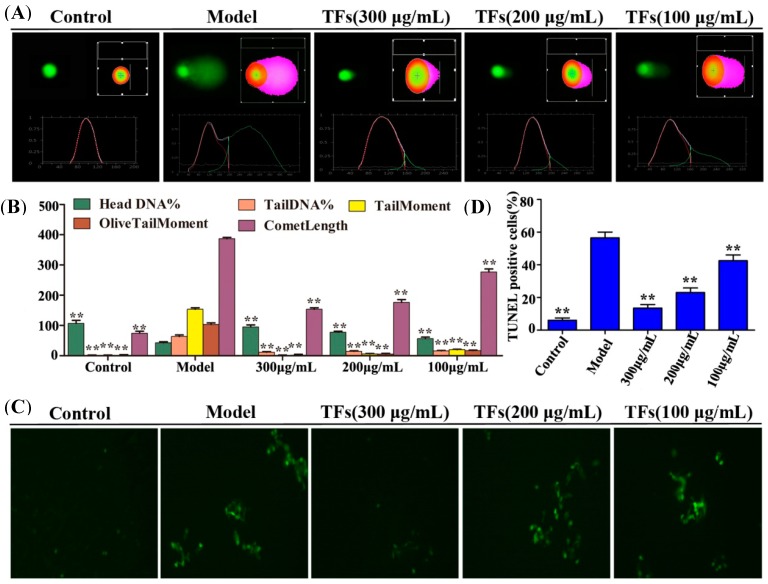
(**A**) The fluorescence images of comet assay photographed by fluorescence microscope (200×, final magnification); (**B**) The data of comet assay; (**C**) TUNEL AI (%) = (TUNEL positive cells/total cells) × 100. (**D**) The number of TUNEL-positive cells per field. Data are presented as mean ± SD (*n* = 5). * *p* < 0.05 and ** *p* < 0.01 compared with model group.

The single cell gel electrophoresis technique was used to detect DNA damage. As shown in Figure 3A,B, the nucleus of the cells was intact and round in shape, and DNA was maintained in the nuclear matrix in the control group. In the model group, DNA damage was detected and the Comet Length was much longer than that of in control group (*p* < 0.01). However, pretreatment with TFs (100, 200 and 300 μg/mL) for 1 h significantly improved DNA damage including Head DNA%, Tail DNA%, Tail Moment and Olive Tail Moment.

### 2.5. TFs Reduced H_2_O_2_-Induced Apoptosis of PC12 Cells

In the present paper, a TUNEL assay was performed to determine the *in situ* DNA fragmentation. As shown in Figure 3C,D, apoptotic cells showing green and brown fluorescence were found. Compared with the control and TFs-treated groups, the numbers of TUNEL-positive cells were increased in the model group with *p* < 0.01.

### 2.6. TFs Inhibited Cytochrome C Release

**Figure 4:**
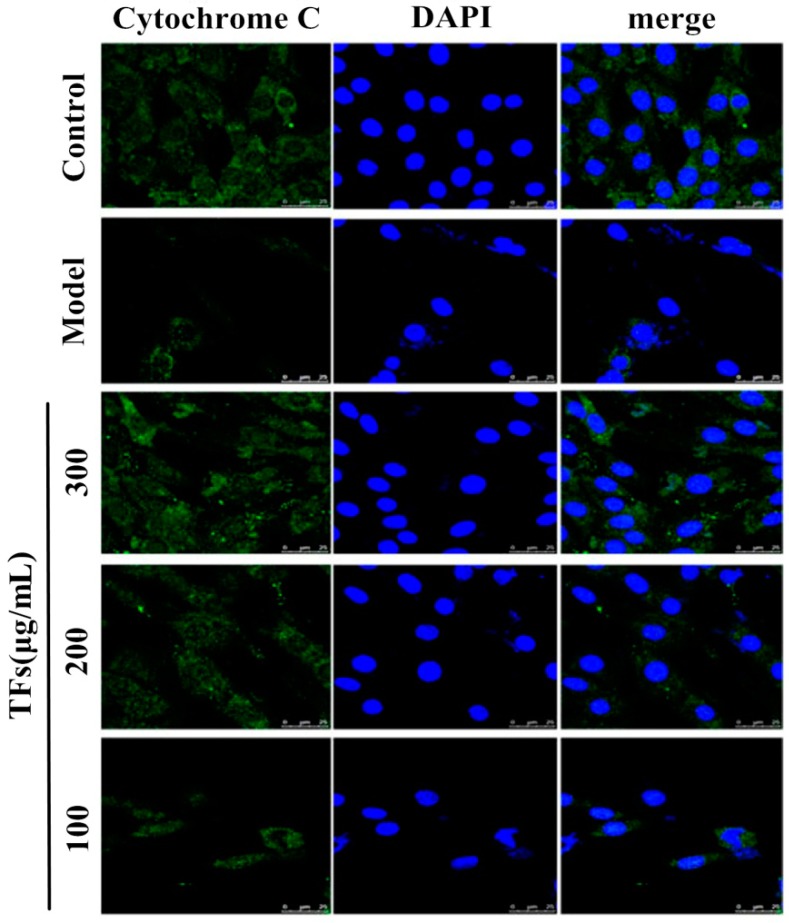
Immunofluorescence of cytochrome C from mitochondrion to cytoplasm (800×, final magnification).

The results shown in Figure 4 indicate that H_2_O_2_ affected the translocation of cytochrome C. A point or massive staining pattern was found in the control group, while a diffuse cytoplasmic staining pattern was found in the model group. However, TFs significantly inhibited the release of cytochrome C from mitochondria to cytoplasm.

### 2.7. Effects of the TFs on the Expressions of H_2_O_2_-Induced Inflammatory Mediators

**Figure 5:**
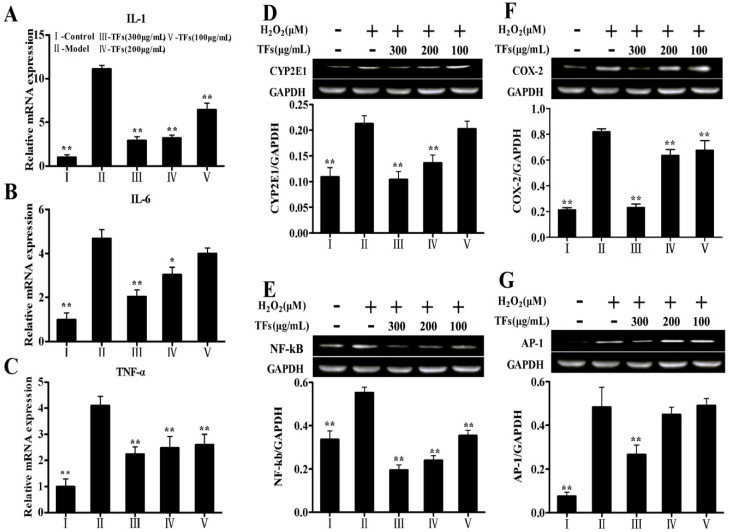
Effects of the TFs on the gene expressions of IL-1 (**A**), IL-6 (**B**), TNF-α (**C**), and protein expressions of CYP2E1 (**D**), NF-kb (**E**), COX-2 (**F**), and AP-1 (**G**). Values are expressed as mean ± SD (*n* = 5). * *p* < 0.05 and ** *p* < 0.01 compared with model group.

As shown in Figure 5, expressions of inflammation-related proteins including IL-1, IL-6, TNF-α, CYP2E1, COX-2, NF-κB and AP-1 were detected, and the results indicated that these expressions were obviously increased compared with the model group, which were significantly down-regulated by the TFs with *p* < 0.01 in the medium or high-dose groups.

### 2.8. Effects of the TFs on the Expressions of H_2_O_2_-Induced Apoptotic Molecules

**Figure 6:**
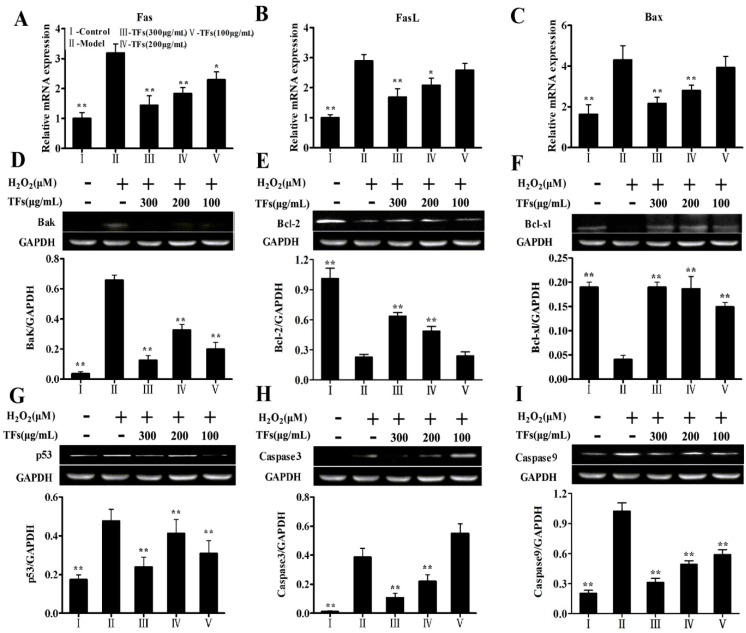
Effects of the TFs on the gene expressions of Fas (**A**), FasL (**B**), Bax (**C**), and protein expressions of Bak (**D**), Bcl-2 (**E**), Bcl-xl (**F**), p53(**G**), caspase-3 (**H**) and caspase-9 (**I**). Values are expressed as mean ± SD (*n* = 5). * *p* < 0.05 and ** *p* < 0.01 compared with model group.

As shown in Figure 6, H_2_O_2_ greatly increased the mRNA expressions of FasL, Fas and Bax (Figure 6A–C) compared with the control group, TFs significantly inhibited their elevations. In addition, some apoptosis-related proteins including Bak, Bcl-2, Bcl-xl, p53, caspase-3 and caspase-9 (Figure 6D–I) were also investigated.

Compared with model group, the expressions of Bak, p53, caspase-3 and caspase-9 were dramatically down-regulated, while the expressions of Bcl-2 and Bcl-xl were significantly up-regulated by 2.33- and 4.5-fold with *p* < 0.01 by TFs (300 μg/mL).

### 2.9. TFs Attenuated H_2_O_2_-Induced the Levels of MAPKs Phosphorylation

**Figure 7:**
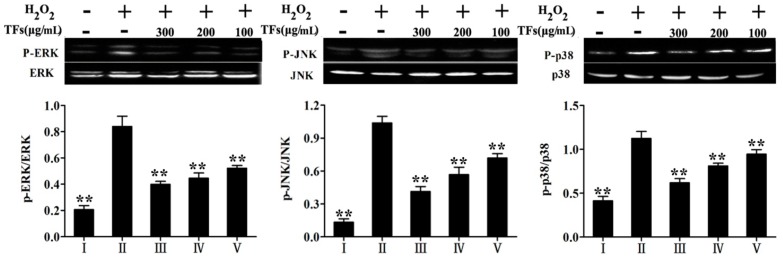
Effects of the TFs on the levels of MAPKs phosphorylation. Datas are presented by phosphorylation/nonphosphorylated. Values are expressed as mean ± SD (*n* = 5). * *p* < 0.05 and ** *p* < 0.01 compared with model group.

The effects of the TFs on the levels of MAPKs phosphorylation were tested in the present work, and the results indicated the levels of p-ERK, p-JNK and p-p38 MAPK activation were significantly increased in the model group by 4.3-, 5.9- and 3.63-fold compared with the ontrol group. However, TFs at the concentration of 300 μg/mL attenuated the expressions by 50.3%, 65.4% and 45.7% compared with the model group, respectively (Figure 7).

### 2.10. Discussion

Oxidative stress-induced cell damage has been implicated in the physiological neurodegenerative disorder process [21]. Currently, many natural products have been widely studied for treatment of neurodegenerative disorders. In our previous works, the TFs from *R. laevigata* Michx fruit down-regulated the protein expressions of CYP2E1, iNOS, NF-kB, Bak, caspase-3, and markedly decreased the mRNA levels of TNF-a, Fas/FasL against against cerebral ischemia-reperfusion injury and carbon tetrachloride (CCl_4_)-induced liver injury, which indicated that the action of TFs may involve oxidative stress reduction and and suppression of inflammation and apoptosis. Furthermore, the tested TFs was orally administered to rats at the doses of 500, 1,000 and 2,000 mg/kg/day, and no toxic signs of the extract at the doses of 500 and 1,000 mg/kg/day were observed [18,19,22]. In the present work, the effects of different concentrations of TFs in the 100–500 μg/mL range to PC12 cells were tested, and we found that the TFs at the dose of 500 μg/mL didn’t affect the viability of PC12 cells. The highest dose of TFs at 300 μg/mL was selected, and we sought to find the effects against H_2_O_2_-induced damage under this condition. The present study confirmed the potent neuroprotective effect of the extract against H_2_O_2_-induced oxidative stress in PC12 cells.

ROS plays an important role in cell apoptosis caused by oxidative stress. Meanwhile, when an increased concentration of intracellular Ca^2+^ caused by oxygen radicals happens, the level of oxidative stress-induced cellular injuries is also accelerated [23,24,25]. As an important member of the ROS family, H_2_O_2_ can cause serious damage via formation of highly toxic hydroxyl radicals [26,27], which can readily attack biological molecules and finally lead to apoptotic or necrotic cell death. In the present work, the level of intracellular ROS in PC12 cells was remarkably increased by H_2_O_2_, however, after treatment with different concentrations of TFs, the cell viability was significantly increased and the ROS level was attenuated. In addition, the increased level of Ca^2+^ in the cytoplasm caused by H_2_O_2_ was also significantly ameliorated by the TFs. These results indicated that the TFs effectively protected PC12 cells against H_2_O_2_-induced cytotoxicity.

CYP2E1 plays an important role in generation of free radicals [18]. In the present work, pretreatment with TFs significantly down-regulated the expression of CYP2E1 in PC12 cells, which indicated that the protective effect of the TFs against H_2_O_2_-induced cell damage may involve affecting the expression of CYP2E1. Furthermore, ROS produced by CYP2E1 and oxidative stress can promote the activation NF-κB, the phosphorylation of ERK and p38 MAPK, and some other biological processes [28].

In an oxidative stress situation, inflammatory reactions can happen with the release of proinflammatory chemokines and cytokines including IL-1β, IL-6 and TNF-α [29]. COX-2, a highly inducible enzyme and a known inflammatory early-response protein [30], is activated by a specific subset of proinflammatory cytokines, which can also be secreted through stimulating HMGB-1. In the current study, we found that the TFs remarkably decreased the mRNA levels of IL-1β, IL-6, TNF-α, and the protein expressions of COX-2 and HMGB-1. Further exploration showed that the TFs reduced the activation of NF-κB and AP-1, which are also the key regulators of some genes involved in inflammation. Our work thus showed that the TFs attenuated inflammation caused by H_2_O_2_.

The fragmentation of DNA is one of the most characteristic phenomena of apoptosis [31]. TUNEL is a method that can visualize the DNA fragmented during apoptosis [32]. In addition, cell apoptosis usually occurs when oxidative stress happens in the intracellular medium. For the apoptotic signal transduction, Fas/FasL is the upstream regulator of caspase-3, and the activity of p53 plays a key role in cell apoptosis, as it is known to promote apoptosis by up-regulating the expression of apoptotic proteins including caspase-9 and caspase-3. The importance of p53 in mediating the cell cycle arrest that occurs following DNA damage has been confirmed [33]. Caspase-3, a key factor of apoptosis in this system, is activated by caspase-9 [34]. The bcl-2 family plays an important role in apoptotic signal transduction by regulating mitochondrial function. Bak and some other proapoptotic proteins can promote cell apoptosis, but Bcl-2 and Bcl-xl are major apoptotic inhibitors [35]. In addition, cytochrome C release is a key event in the activation of caspase-3, a pivotal downstream step in apoptosis initiation [36,37]. In the present paper, we found that TFs significantly decreased the protein expressions of Bak, caspase-3, p53, caspase-9, and the mRNA expressions of Bax, Fas and FasL, while increased the levels of Bcl-2 and Bcl-x. These findings indicated that the TFs suppressed cell apoptosis caused by H_2_O_2_.

When the MAPKs are activated, they can phosphorylate their specific cascade proteins and therefore control many cellular activities, including cell proliferation, differentiation and cell death [38,39]. Therefore, we continued to study the underlying signaling pathways by detecting the involvement of ERK, p38 and JNK MAPK pathways in the protective effects of TFs against H_2_O_2_-induced PC12 cells injury. The results showed that the phosphorylation levels of JNK, ERK and p38 MAPK were markedly increased in the model group, which were all significantly attenuated by the extract. This suggested that the TFs affected the MAPK signaling pathway against H_2_O_2_-induced PC12 cell injury. Taken together, cell apoptosis caused by ROS-induced oxidative stress was found, and the TFs scavenged ROS to reduce the apoptosis.

## 3. Experimental

### 3.1. Chemicals and Reagents

Analytical grades of chemicals including 3-(4,5-dimethylthiazol-2-yl)-2,5-diphenyltetrazolium bromide (MTT), penicillin, dimethyl sulfoxide (DMSO), hydrogen peroxide (H_2_O_2_), streptomycin, hydroxyethyl piperazine ethanesulfonic acid (HEPES), poly-L-lysine, DCFH-DA and 4,6-damidino-2-phenylindole (DAPI) were all purchased from Sigma Chemical Co. (St. Louis, MO, USA). Flou-3/AM was provided by Dojindo Molecular Technologies, Inc (Kumamoto, Japan). *In situ* Cell Death Detection Kit were obtained from Roche Diagnostics (Mannheim, Germany). Acridine orange (AO) and ethidium bromide (EB) fluorescent dyes were purchased from Nanjing KeyGen Biotech. Co. Ltd. (Nanjing, China).

### 3.2. Herbal Material and Preparation of the Total Flavonols

The herbal material *Rosa laevigata* Michx fruit was purchased from Yunnan Qiancaoyuan Pharmaceutical Company Co. Ltd. (Yunnan, China), and identified by Dr. Yunpeng Diao (Dalian Medical University, Dalian, China). A voucher specimen (DLMU, JYZ080426) was deposited in the Herbarium of the College of Pharmacy of Dalian Medicinal University. The total flavonols (TFs) from *R. laevigata Michx* fruit was prepared and analyzed according to the described methods reported in our previous study, in which three major chemicals including quercetin, kaempferide and isorhamnetin were identified and their contents were also determined at the levels of 3.11%, 2.72% and 1.49%, respectively, by high-performance liquid chromatography (HPLC) [40].

### 3.3. Cell Culture

Rat pheochromocytoma undifferentiated PC12 cells with the characteristics and function of neuron cells and the ability to split and differentiate, which have been widely used as a cell line that provides a useful model system for neurological and neurochemical studies, were obtained from the Institute of Biochemistry Cell Biology (Shanghai, China). The cells were maintained in DMEM containing 10% FBS, 100 U/mL penicillin and 100 U/mL streptomycin at 37 °C with 5% CO_2_. In all experiments, the culture medium was altered once every three days, and the cells were allowed to adhere and grow for 24 h before treatment.

### 3.4. Assay of Cell Viability

In brief, the PC12 cells were seeded in 96-well plates at 10^5^ cells/well with the different concentrations of TFs for 1 h before being exposured to 400 μM H_2_O_2_ for 4 h at 37 °C. Then, MTT (10 μL, 5 mg/mL) solution was added to each culture well for further incubation. After 4 h, the culture medium was removed and the formazan crystals were dissolved by addition of 150 μL DMSO to each well with vigorously shaking the plate to ensure complete solubilization. Finally, formazan absorbance was assessed by a POLARstar OPTIMA multi-detection microplate reader (BioRad, San Diego, CA, USA) at 490 nm.

### 3.5. AO/EB and DAPI Staining

PC12 cells cultured in 6-well plates at the density of 10^6^ cells/well were pretreated as described above. The cells were washed twice with cold PBS, and the mixture containing same volume of AO (100 μg/mL in PBS) and EB (100 μg/mL in PBS) was put onto the cells, and then the images of the cells were obtained by using an inverted fluorescence microscope (Olympus BX63, Tokyo, Japan).

PC12 cells cultured in 6-well plates at the density of 10^6^ cells/well were pretreated as described above. The cells were washed twice with PBS and stained with DAPI (1 μg/mL) solution for 10 min at 37 °C, then washed twice with PBS. Finally, the cells were photographed by using an inverted fluorescence microscopy (Olympus BX63).

### 3.6. Single Cell Gel Electrophoresis Assay

Single cell gel electrophoresis (SCGE) assay was carried out to detect H_2_O_2_- induced DNA damage. After being pretreated, the comet assay was performed under the manufacturer’s instructions (Cell Biolabs Inc, San Diego, CA, USA). The images of the cells were obtained by using a fluorescence microscope (Olympus BX63). Eventually, at least 50 cells from each of the three repeated wells were randomLy selected and analyzed with the Comet Assay Software Project (CASP).

### 3.7. TUNEL Assay

The PC12 cells were seeded in six-well plates and incubated overnight. After being pretreated, TUNEL assay was performed according to the manufacturer’s instructions. At last, the cells were obtained by using a fluorescence microscope (Olympus BX63). The percentages of TUNEL positive cells were counted by LSM510 Expert Mode SP2 software (Zeiss, Jena, Germany).

### 3.8. Detection of Cytochrome C Release

The PC12 cells were plated in six-well incubated overnight to detect cytochrome C release. After pretreatment, the cells were washed with PBS, fixed with 4% paraformaldehyde for 15 min at 4 °C, washed with PBS again, and then permeabilized with 0.2% Triton-100 for 8 min. Non-specific binding was blocked by incubating cells in 3% BSA for 45 min at 37 °C, and the cells were hatched with the primary antibody overnight at 4 °C. Then, the plates were washed twice with PBS. The cells were incubated with secondary antibody for 1 h at 37 °C, washed with PBS, then stained with DAPI (1 μg/mL) for 5 min. The images of the cells were photographed by using a laser scanning confocal microscope (TCS SP5, Leica, Wetzlar, Germany).

### 3.9. Detection of Intracellular ROS Accumulation and Ca^2+^ Release

The PC12 cells were plated in 12-well plates at the density of about 5 × 10^5^ cells/well and treated as described above. The cells were collected and then re-suspended in 500 μL of DCFH-DA (10 μM) for detecting of ROS and in 500 μL of Fluo-3/AM (2.5 μM) for detection of Ca^2+^ release, which were all analyzed by flow cytometry (FACSCalibur, Becton Dickson, San Diego, CA, USA).

### 3.10. Transmission Electron Microscope (TEM) Assay

The PC12 cells were seeded in six-well plates and incubated overnight. After being pretreated as described above, the cells were fixed in 2% glutaraldehyde overnight at 4 °C, and the samples were implemented as previously described [41]. Finally, the stained ultramicrotomies were photographed by using a transmission electron microscope (JEM-2000EX, JEOL, Sagamihara, Japan).

### 3.11. Quantitative Real-time PCR Analyses

Total RNA samples from the PC12 cells were extracted by using RNAiso Plus reagent following the manufacturer’s protocol. Reverse transcripttion polymerase chain reaction (RT-PCR) was performed using PrimeScript^®^ RT reagent Kit following the manufacturer’s instructions with a TC-512 PCR system (TECH-NE, Stone, UK). The levels of mRNA expression were quantified by real-time PCR with SYBR^®^ PremixEx Taq™II (Tli RNaseH Plus) and ABI 7,500 Real Time PCR System (Applied Biosystems, Carlsbad, CA USA), and the data was analyzed by System SDS software (Applied Biosystems). The sequences of the primers were as follows: GAPDH: forward, 5'-GGCACAGTCAAG- GCTGAGAATG-3', reverse, 5'-ATGGTGGTGAAGACGCCAGTA-3';IL-1β, forward, 5'-CCCTGAACTCAACTGTGAAATAGCA-3', reverse, 5'-CCCAAGTCAAGGGCTTGGA A-3'; IL-6, forward, 5'-ATTGTATGAACAGCGATGATGCAC-3', reverse, 5'-CCAGGTAGAAACGG AACTCCAGA-3'; TNF-α, forward, 5'-TCAGTTCCATGGCCCAGAC-3', reverse, 5′-GTTGTCTTTG AGATCCATGCCATT-3′; Fas, forward, 5'-CACAGCATTCAGTCCTATCCACAGA-3', reverse, 5′-C ACAGCCAACCAGATGCTTCA-3'; FasL, forward, 5'-CACCAACCACAGCCTTAGAGTATCA-3', reverse, 5'-CACTCCAGAGATCAAAGCAGTTCC-3'; Bax, forward, 5'-CGAATTGGCGATGAACT GGA-3', reverse, 5'-CAAACATGTCAGCTGCCACAC-3'. A no-template control was analyzed in parallel for each gene, and GAPDH gene was selected as the housekeeping gene in our study. Finally, the unknown template was calculated through the standard curve for quantitative analysis.

### 3.12. Western Blotting Assay

The PC12 cells were seeded in six-well plates and incubated overnight. After being pretreated with the different concentrations of TFs for 1 h before exposure to 400 μM H_2_O_2_ for 2 h at 37 °C, total proteins from different groups were extracted by using cell lysis buffer containing phenylmethane -sulfonyl fluoride (PMSF). Lysates were centrifuged at 12,000 *g* for 10 min at 4 °C and the total protein was obtained. An aliquot (50 μg protein) was loaded onto a 12% SDS-PAGE gels and separated electrophoretically. Then the target proteins were transferred to a PVDF membrane (Millipore, Billerica, MA, USA). After blocking the PVDF membrane in 5% dried skim milk for 3 h at room temperature, the membrane was incubated overnight at 4 °C with primary antibodies (Table 1). Then the membrane was incubated at room temperature for 2 h with horseradish peroxidase-conjugated goat anti-rabbit IgG or goat anti-mouse IgG antibodies at a 1:2,000 dilution. Protein detection was performed based on an enhanced chemiluminescence (ECL) method and photographed by using a BioSpectrum Gel Imaging System (UVP, Upland, CA, USA). In order to eliminate the variations, data were adjusted to GAPDH expression: IOD of objective protein *versus* IOD of GAPDH expression.

**Table 1:** The antibodies used in the present work

Antibody	Source	Dilutions	Company
GAPDH	rat	1:5,000	Proteintech Group, Chicago, IL, USA
CYP2E1	rabbit	1:1,000	Proteintech Group, Chicago, IL, USA
COX-2	rabbit	1:1,000	Proteintech Group, Chicago, IL, USA
HMGB-1	rabbit	1:1,000	Proteintech Group, Chicago, IL, USA
NF-κB	rabbit	1:1,000	Proteintech Group, Chicago, IL, USA
AP-1	rabbit	1:1,000	Proteintech Group, Chicago, IL,.USA
Bak	rabbit	1:1,000	Proteintech Group, Chicago, IL,.USA
Bcl-2	rabbit	1:1,000	Proteintech Group, Chicago, IL, USA
Bcl-x	rabbit	1:1,000	Proteintech Group, Chicago, IL, USA
p53	rabbit	1:1,000	Proteintech Group, Chicago, IL, USA
Caspase-3	rabbit	1:1,000	Proteintech Group, Chicago, IL, USA
Caspase-9	rabbit	1:1,000	Proteintech Group, Chicago, IL, USA
p-p38	rabbit	1:1,000	Bioworld Technology, St. Louis Park, MN, USA
p-38	rabbit	1:1,000	Bioworld Technology, St. Louis Park, MN, USA
p-ERK	rabbit	1:1,000	Bioworld Technology, St. Louis Park, MN, USA
ERK	rabbit	1:1,000	Bioworld Technology, St. Louis Park, MN, USA
p-JNK	rabbit	1:1,000	Bioworld Technology, St. Louis Park, MN, USA
JNK	rabbit	1:1,000	Bioworld Technology, St. Louis Park, MN, USA

### 3.13. Statistical Analysis

All values for each group were given as mean and standard deviation (SD). The data were analysed by one-way analysis of variance (ANOVA) coupled with LSD in *Post Hoc* Multiple Comparisons using the SPSS Statistics 15.0 (IBM, New York, NY, USA) package. A value of *p* < 0.05 was considered statistically significant.

## 4. Conclusions

In conclusion, the TFs have a potent protective effect against H_2_O_2_-induced PC12 cell damage via attenuating oxidative stress, inflammation and apoptosis, and could be developed as a new food application to protect from neurodegenerative disorders in the future. Of course, the chemical compositions, in depth mechanisms, drug-targets and applications of the TFs need further investigations.
