# Correction: Liu, M., et al. Potent Effects of Flavonoid-Rich Extract from *Rosa laevigata* Michx Fruit against Hydrogen Peroxide-Induced Damage in PC12 Cells via Attenuation of Oxidative Stress, Inflammation and Apoptosis. *Molecules* 2014, *19*, 11816–11832.

**DOI:** 10.3390/molecules24234228

**Published:** 2019-11-20

**Authors:** Min Liu, Youwei Xu, Xu Han, Chen Liang, Lianhong Yin, Lina Xu, Yan Qi, Yanyan Zhao, Jinyong Peng, Changkai Sun

**Affiliations:** 1 College of Pharmacy, Dalian Medical University, Western 9 Lvshunnan Road, Lvshunkou District, Dalian 116044, China; zhengyi_2019@163.com (M.L.); Youweixu0112@163.com (Y.X.); Xuhan0118@163.com (X.H.); Lianhongyin0112@163.com (L.Y.); Linaxu0112@163.com (L.X.); Yanqi0118@163.com (Y.Q.); Yanyanzhao_2009@126.com (Y.Z.); 2 College of Basic Medical Sciences, Dalian Medical University, Western 9 Lvshunnan Road, Lvshunkou District, Dalian 116044, China; Qiaoyujie1993@163.com; 3 Research Institute of Integrated Traditional and Western Medicine of Dalian Medical University, Dalian 116044, China; 4 Liaoning Provincial Key Laboratory of Brain Diseases and Institute of Medical Education, Western 9 Lvshunnan Road, Lvshunkou District, Dalian 110644, China

During the course of a review of our publication, an error in the title paper [1] has come to our attention. This error affects the flow cytometry data for the model group presented in Figure 2A. We provide the correct figure below. The data have been reanalyzed and have been determined to have no influence on the reported results. 

All co-authors agree with the content of this Correction and wish to apologize for any inconvenience to the readers resulting from this error.

## Figures and Tables

**Figure 2:**
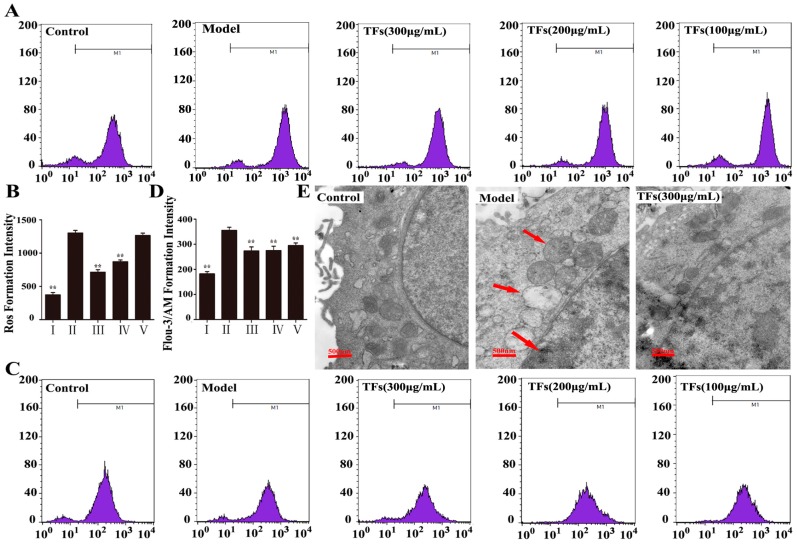
ROS generation detected by flow cytometry (**A**,**B**); The level of Ca^2+^ detected by flow cytometry (**C**,**D**); Protective effect of the TFs on the ultra-structure of PC12 cells (40,000×, final magnification) (**E**). Data are presented as mean ± SD (*n* = 5). * *p* < 0.05 and ** *p* < 0.01 compared with model group. The arrows pointed cytoplasmic vacuoles, chromatin condensation and mitochondrial swelling of the cells treated by H_2_O_2_.
